# Clinical characteristics of branch retinal vein occlusion with increased retinal haemorrhage during treatment for macular oedema

**DOI:** 10.1038/s41598-020-67395-7

**Published:** 2020-06-24

**Authors:** You Hyun Lee, Yu Cheol Kim

**Affiliations:** Department of Ophthalmology, Keimyung University Dongsan Medical Center, #1035 Dalgubeoldae-ro, Dalseo-gu, Daegu, 42601 Korea

**Keywords:** Diseases, Risk factors, Signs and symptoms

## Abstract

This study was performed to evaluate clinical characteristics of branch retinal vein occlusion (BRVO) patients with increased retinal haemorrhage during macular oedema (MO) treatment. Patients were divided into increased and non-increased retinal haemorrhage groups. The former group was sub-divided based on the degree of increase: < 50% or ≥ 50%. Baseline characteristics, clinical data, and best vision achieved before and after increased retinal haemorrhage were assessed. Sixty-eight eyes of 68 patients were included. Forty-six eyes were non-increased, 11 eyes experienced < 50% increase in retinal haemorrhage, and 11 eyes had ≥ 50% increase. Ischaemic BRVO was related to increased haemorrhage based on the multivariate analysis. The ≥ 50% increase group exhibited higher baseline central subfield macular thickness (CSMT), poorer baseline best corrected visual acuity (BCVA), and longer mean periods between the final intravitreal injections and the time increased retinal haemorrhages were observed, compared to the < 50% group. The best vision achieved before and after increased haemorrhage was significantly worse in the ≥ 50% group. In conclusion, the ischaemic type of BRVO is related to increased retinal haemorrhage during MO treatment, and a ≥ 50% increase in haemorrhages is associated with higher CSMT and poorer BCVA at baseline, with poor visual gain.

## Introduction

Branch retinal vein occlusion (BRVO) is the second most common retinal vascular disorder after diabetic retinopathy with an incidence of 0.5–1.2%^1,2^. BRVO patients present with varying degrees of retinal haemorrhage, tortuous retinal vessels, retinal ischaemia, and macular oedema (MO). Furthermore, the MO associated with BRVO is known to be the most common cause of visual impairment and develops in up to 60% of cases^3^. However, an evidence-based systemic review of the natural history of 1,608 cases of BRVO showed that only 18–41% of baseline MO cases resolved within 24 months^1^.

For this reason, most previously reported BRVO studies have focused on the treatment of MO. Laser photocoagulation, intravitreal injection of steroids or anti-vascular endothelial growth factor (VEGF) agents such as bevacizumab, ranibizumab, or aflibercept, and vitrectomy have been used for the treatment of MO in cases of BRVO^4–7^. It is known that VEGF is also related to persistent haemorrhages in RVO, which can be reduced by intravitreal anti-VEGF treatments^8,9^. However, to the best of our knowledge, there have been no studies assessing increased retinal haemorrhages during the treatment of MO secondary to BRVO.

Therefore, we aimed to evaluate the clinical characteristics of BRVO in patients experiencing increased retinal haemorrhages during MO treatment.

## Methods

### Study design and subjects

This study was designed as a retrospective, comparative case series conducted in a single hospital. The study adhered to the tenets of the Declaration of Helsinki and was approved by the institutional review board of Keimyung University Dongsan Hospital (IRB no. 2020-03-018). The patients signed informed consent for the use of their data. We retrospectively reviewed the electronic medical records (EMR) of BRVO patients who were followed up for at least 6 months after MO treatment from January 2016 to December 2018. MO was defined as central subfield macular thickness (CSMT) ≥ 300 µm. The exclusion criteria were as follows: severe media opacities that prevented the interpretation of images, a history of pars plana vitrectomy or cataract surgery within the 6 months preceding the start of the trial, the presence of other retinal diseases that could cause decreased BCVA or MO, such as epiretinal membrane, a grade exceeding severe non-proliferative diabetic retinopathy, rhegmatogenous retinal detachment, age-related macular degeneration, and uveitis. Patients who did not show increase in retinal haemorrhages during the follow-up period and who were followed up for at least 6 months were allotted to the non-increased group. Increased retinal haemorrhage was defined as an increase, detectable by the human eye, based on the fundus colour images; patients who were followed up for at least 6 months after the detection of increased haemorrhage were allotted to the increased group. The increase in retinal haemorrhages was measured using the National Institutes of Health's image-analysis software (ImageJ 1.44p; National Institutes of Health, Bethesda, MD, USA)^10,11^.

**Figure 1:**
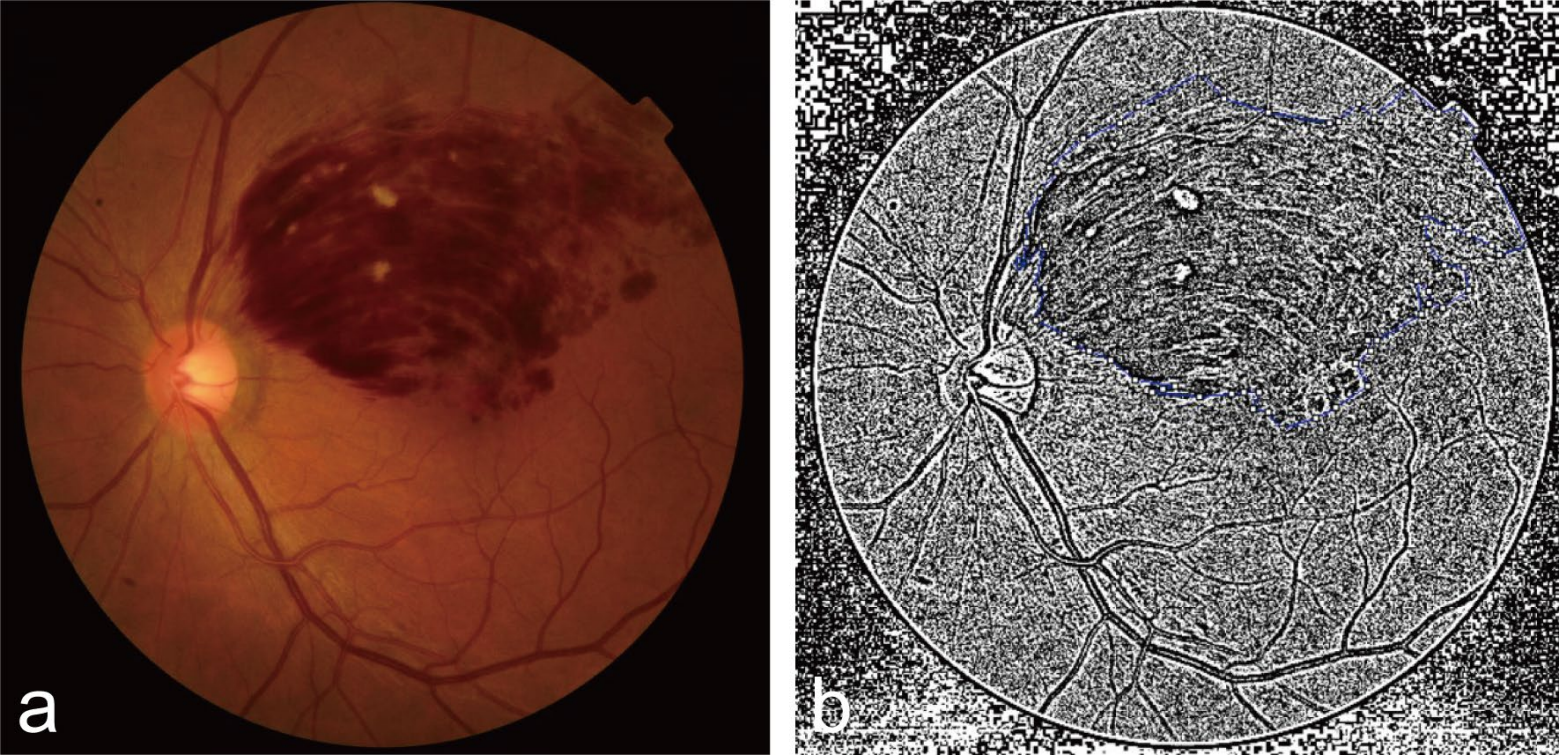
Measurement of amount of retinal haemorrhage. (**a**) A fundus colour photography. (**b**) A grayscale version of the fundus photograph transformed using the automatic thresholding method (local Otsu with a radius of 18 pixels) in Image J software version 1.44p (https://imagej.nih.gov/ij/). The perimeter defining the area was traced based on the fundus colour photograph.

**Figure 2:**
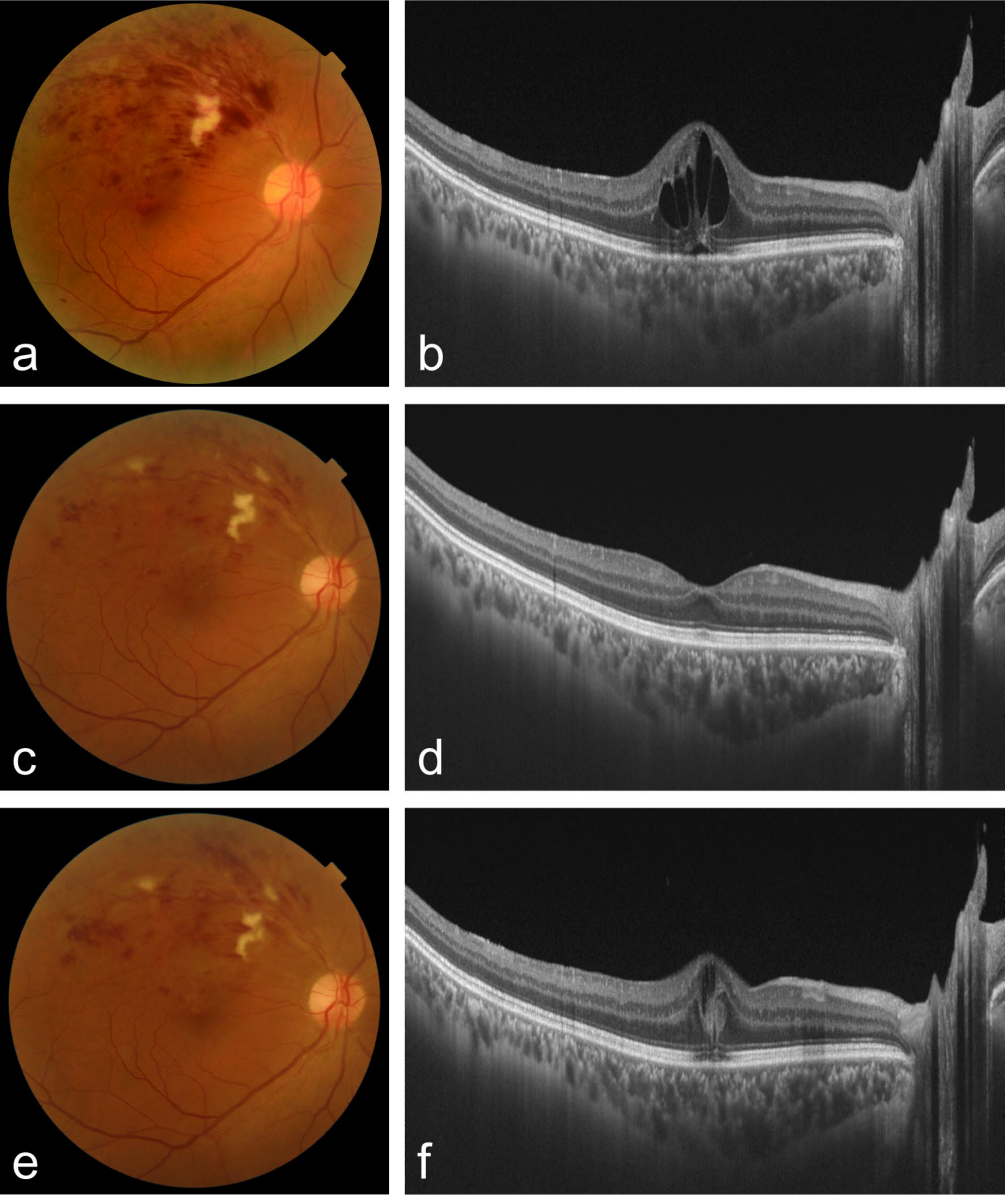
Fundus colour photography and optical coherence tomography of < 50% increased retinal haemorrhages. (**a**,**b**) Initial visit. (**c**,**d**) A visit before the increase in retinal haemorrhages. (**e**,**f**) A visit of increase in retinal haemorrhages.

**Figure 3:**
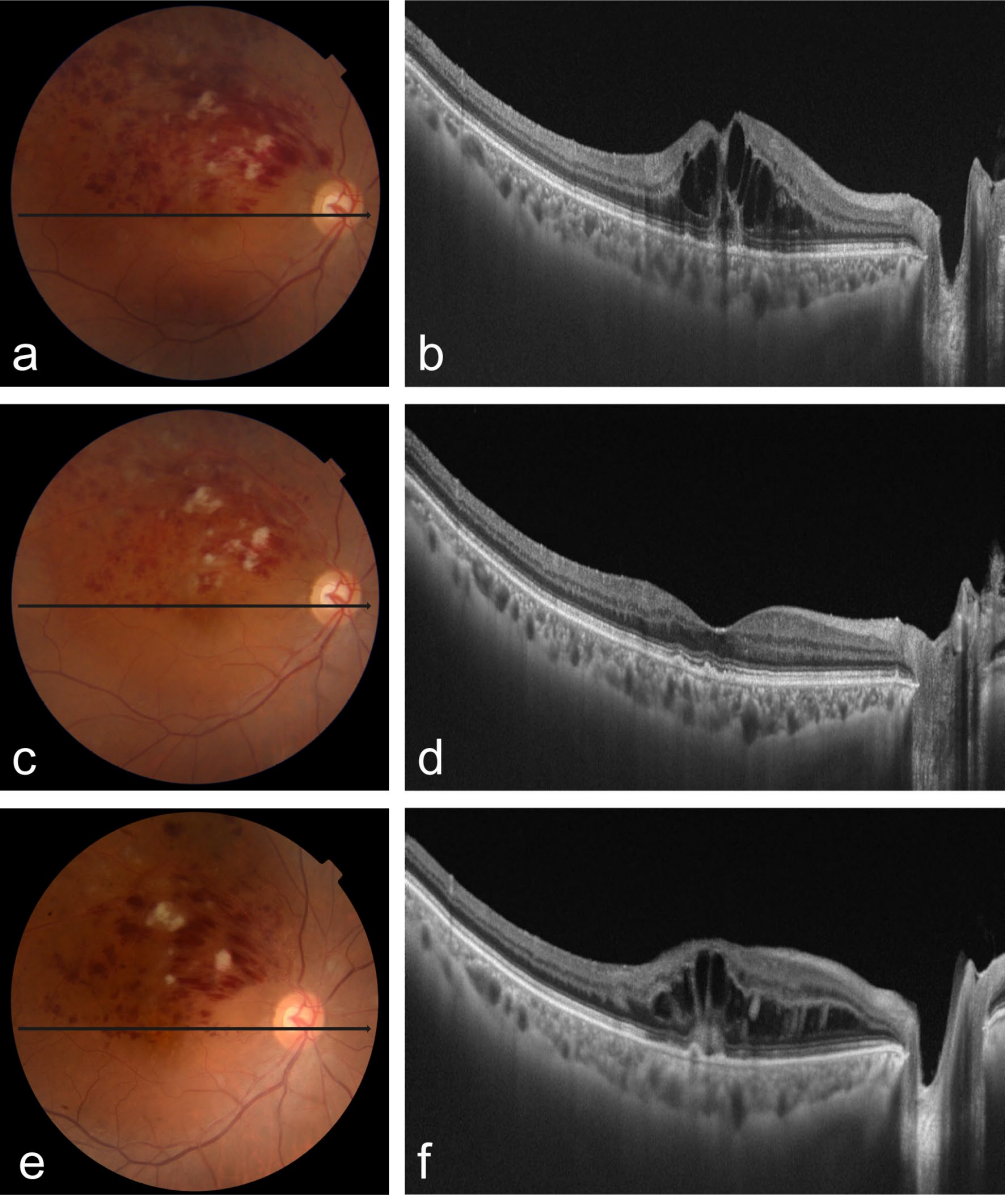
Fundus colour photography and optical coherence tomography of ≥ 50% increased retinal haemorrhages. (**a**,**b**) Initial visit. (**c**,**d**) A visit before the increase in retinal haemorrhages. (**e**,**f**) A visit of increase in retinal haemorrhages.

Colour images were obtained at each of three timepoints (timepoint 1: initial baseline; timepoint 2: a visit prior to the increase in retinal haemorrhage; timepoint 3: the day the increased retinal haemorrhage was detected). The images were converted into grayscale and were thresholded (local Otsu with a radius of 18 pixels) using ImageJ. The perimeter of the retinal haemorrhagewas drawn by a clinician (YHL) using the polygon selection tool based on the fundus colour image, and the number of pixels in this area with a value of zero was counted (Fig. 1). The ratio of the percent increase in the count of zero value pixels at timepoint 3 (count of zero value pixels at timepoint 3 minus count at timepoint 2) to the percent decrease at timepoint 2 (count at timepoint 1 minus count at timepoint 2) was determined. The increased retinal haemorrhage group was further divided into two groups (< 50%, Fig. 2, and ≥ 50% increase, Fig. 3) for subgroup analysis.

### Clinical data collection

At the initial visit, demographic characteristics, including the age, sex, laterality, presence of hypertension, diabetes, cerebrovascular attack, and hyperlipidaemia were assessed. Also, the baseline logMAR BCVA, intraocular pressure (IOP) measured by a non-contact tonometer (Canon TX-20, Canon Inc, Kanagawa, Japan), and spherical equivalent values were recorded. The location of the BRVO was categorized as either superior or inferior. The major type of BRVO and the macular type of BRVO were categorized based on the site of where the retinal vascular occlusion was seen in the fundus colour images; the major type was defined as an occlusion of a temporal arcade vein or branch extending to the peripheral retina beyond the retinal vascular arcades; the macular type was defined as an occlusion confined between the superior and inferior retinal temporal vascular arcades^12,13^. The baseline CSMT was defined as the average thickness at the centre of a 1 mm diameter circle of the Early Treatment Diabetic Retinopathy Study grid, while subfoveal choroidal thickness (SCT) was defined as the thickness from the outer line of the retinal pigment epithelium to the chorioscleral interface. The CSMT and SCT were measured using OCT (DRI OCT-1; Topcon, Tokyo, Japan), and all patients underwent fluorescein angiography (FA;HRA-2; Heidelberg Engineering, Heiderberg, Germany) to identify the occlusion site. The occlusion distance was defined as the distance (µm) between the optic disc margin and the occlusion site. Ischaemic BRVO was defined as a nonperfusion exceeding a five-disc diameter based on FA. The treatment of MO secondary to BRVO included an intravitreal injection of either bevacizumab (1.25 mg/0.05 mL) or triamcinolone (4.0 mg/0.05 mL). The risk of glaucoma and the possibility of systemic side effects were considered in the choice of the intravitreal injection and performed in the pro re nata regimen. The patients’ follow-up interval was between 1 and 3 months.

When an increase in retinal haemorrhages was observed, the BCVA, IOP, CSMT, and SCT were assessed every 3 months from the date of the event.

### Statistical methods

Data were calculated as means ± standard deviation (SD), or n (%; e.g., number of eyes). Statistics were performed using Statistical Package for the Social Sciences (SPSS) version 12.0 (IBM, Chicago, IL, USA). The between-group differences in age, BCVA, IOP, CSMT, SCT, and occlusion distance were compared between groups using independent t tests. The categorical variables, such as sex, affected eye, the presence of systemic diseases, the location of the BRVO, the type of BRVO (ischaemic versus non-ischaemic, and major type versus macular type), and baseline treatment (bevacizumab versus triamcinolone) were compared using chi square tests. Binary logistic regression analysis was used to identify factors associated with increased retinal haemorrhages. The subgroup analysis to compare differences between the those with increased retinal haemorrhages of < 50% and ≥ 50% was performed using Mann–Whitney U tests for continuous variables and Fisher’s exact test for categorical variables. The comparison of best vision achieved before and after an increase in retinal haemorrhages was performed using Wilcoxon signed-rank tests.

## Results

### Demographic characteristics

A total of 68 eyes of 68 patients were included in this study. The number of increased retinal hemorrhages was noted in 22 eyes of 22 patients. Among the increasedhaemorrhage group, an increase of ≥ 50% was visible in 11 eyes of 11 patients. Demographic data of patients displaying an increase in retinal haemorrhages is shown in Table 1. The mean age was 68 ± 10 years in the increased haemorrhage group and 61 ± 13 years in the non-increased group; there was a significant difference in age between the two groups (*p* = 0.032). The affected eye, sex, and systemic diseases such as hypertension, diabetes mellitus, hyperlipidaemia, and cerebrovascular accident did not differ significantly between the groups (*p* > 0.05).

**Table 1:** Demographics of increased versus non-increased retinal haemorrhage groups in branch retinal vein occlusion patients.

**Parameters**	**Increased group (n = 22)**	**Non-increased group (n = 46)**	***P value***
Age in years, mean (SD)	68 (10)	61 (13)	0.032*
Sex, n (%) male	10 (43.5%)	20 (45.5%)	0.878
Laterality, n (%) right eye	14 (63.6%)	23 (50.0%)	0.291
Diabetes, n (%)	4 (18.2%)	6 (13.0%)	0.717
Hypertension, n (%)	6 (27.3%)	18 (39.1%)	0.421
Hyperlipidaemia, n (%)	2 (9.1%)	2 (4.3%)	0.590
Cerebrovascular accident, n (%)	0 (0.0%)	1 (2.2%)	1.000

*Statistically significant as calculated using independent two-sample t test or Pearson’s chi square test.

### Clinical characteristics of the increased and non-increased haemorrhage groups

A comparison of the clinical characteristics between the two groups is shown in Table 2. A longer total follow-up period was observed in the increased group (14 ± 3 months); however, there was an insignificant difference when compared with the non-increased group (13 ± 3 months, *p* = 0.137). The total number of intravitreal injections showed an insignifanct difference between the increased and non-increased groups (4 ± 1 versus 3 ± 1, *p* = 0.093, respectively). The baseline BCVA was not significantly different (increased group versus non-increased group, 0.71 ± 0.25 logMAR versus 0.60 ± 0.33 logMAR, *p* = 0.158). The difference in the mean IOP was not significant between the increased group (14.36 ± 2.63 mmHg) and the non-increased group (13.72 ± 2.15 mmHg, *p* = 0.285). The difference in the mean spherical equivalent was also not significant between the increased group (1.19 ± 0.74) and the non-increased group (1.82 ± 2.44, *p* = 0.146). The mean CSMT was significantly higher in the increased group (512.95 ± 103.40 μm) than in the non-increased group (445.43 ± 134.05 μm, *p* = 0.041). The mean SCT did not significantly differ between the two groups (203.86 ± 82.09μmin the increased group versus 244.22 ± 105.05 μm in the non-increased group, *p* = 0.120). The percentages of superior segment involvement, macular type, and initial intravitreal bevacizumab injection showed no differences between the two groups (59.1% versus 82.6%, 22.7% versus 34.8%, and 86.4% versus 89.1%, respectively, *p* > 0.05). However, the ischaemic type was significantly more visible in the increased group (54.5% versus 15.2%). Moreover, the occlusion distance from the optic disc margin was significantly shortened in the increased retinal haemorrhage group (794.41 ± 521.21 μm versus 1,134.17 ± 703.27 μm).

**Table 2:** Comparison of clinical characteristics between the increased and non-increased retinal haemorrhage groups in branch retinal vein occlusion patients.

**Parameters**	**Increased group (n = 22)**	**Non-increased group (n = 46)**	***P value***
Area, superior, n (%)	13 (59.1%)	38 (82.6%)	0.070
Type of BRVO, ischaemic type, n (%)	12 (54.5%)	7 (15.2%)	0.001*
Type of BRVO, macular, n (%)	5 (22.7%)	16 (34.8%)	0.405
Initial therapy, bevacizumab, n (%)	19 (86.4%)	41 (89.1%)	0.707
Total follow-up period, months, mean (SD)	14 (3)	13 (3)	0.137
Total number of intravitreal injections, mean (SD)	4 (1)	3 (1)	0.093
Baseline best corrected visual acuity, logMAR (SD)	0.71 (0.25)	0.60 (0.33)	0.158
Baseline intraocular pressure, mmHg, mean (SD)	14.36 (2.63)	13.72 (2.15)	0.285
Baseline spherical equivalent, mean (SD)	1.19 (0.74)	1.82 (2.44)	0.146
Baseline central subfield macular thickness, μm, mean (SD)	512.95 (103.40)	445.43 (134.05)	0.041*
Baseline subchoroidal thickness, μm, mean (SD)	203.86 (82.09)	244.22 (105.05)	0.120
Occlusion distance from optic disc margin, μm, mean (SD)	794.41 (521.21)	1,134.17 (703.27)	0.048*

*BRVO* branch retinal vein occlusion.

*Statistically significant as calculated using independent two-samples t test or Pearson’s chi square test.

### Analysis of the factors associated with increased retinal haemorrhages

In binary logistic regression analysis, the ischaemic type of BRVO was the only factor that was significantly associated with increased retinal haemorrhages during the treatment of MO (*p* = 0.007; Table 3).

**Table 3:** Multivariate analysis of possible factors associated with increased retinal haemorrhages in branch retinal vein occlusion patients.

**Parameters**	**Adjusted OR**	**95% CI**	***P value***
Age			0.139
Baseline central subfield macular thickness			0.107
Ischaemic type	8.879	1.855–19.942	0.003*
Occlusion distance from the optic disc margin			0.382

Variables with *p* < 0.05 from the univariate analyses were selected into the binary logistic regression model.

*OR* odds ratio, *CI* confidence interval.

*Statistically significant as calculated using binary logistic regression analysis with backward elimination (R^2^ = 0.255, *P* = 0.717 by the Hosmer–Lemeshow test for goodness of fit).

### Subgroup analysis based on the increased amount of retinal haemorrhages

The demographics of increased retinal haemorrhages between the < 50% and the ≥ 50% increase groups are shown in Table 4. There were no significant differences between the two groups (*p* > 0.05). The comparison of clinical characteristics between the two groups is shown in Table 5. The average percent increases in the amount of retinal haemorrhages were 10.18 ± 7.32 in the < 50% group and 73.76 ± 13.37 in the ≥ 50% group (*p* < 0.001). The interval between the baseline and the time the increased retinal haemorrhages were detected showed insignificant difference between the < 50% group (5 ± 2 months) and the ≥ 50% group (7 ± 2 months, *p* = 0.088). The mean period between the last intravitreal injection and the time the increased retinal haemorrhages were detected was 59.27 ± 10.53 days in the < 50% group and 103.27 ± 26.85 days in the ≥ 50% (*p* < 0.001). The baseline BCVA was significantly worse in the ≥ 50% group (0.88 ± 0.15 logMAR) than in the < 50% group (0.60 ± 0.23 logMAR, *p* = 0.015). Moreover, a thicker baseline CSMT was observed in the ≥ 50% group compared to the < 50% group (561.46 ± 69.89 versus 461.82 ± 106.17 μm, *p* = 0.013). As the baseline CSMT showed significant difference between the two groups, the ratio of the CSMT at the date the increased retinal haemorrhages were observed and the baseline CSMT was also assessed; the ratio was also significantly higher in the ≥ 50% group (78.03 ± 13.30 versus 66.02 ± 13.57, *p* = 0.019). Other characteristics such as the superior segment involvement, ischaemic type, macular type, initial intravitreal bevacizumab injection, baseline IOP, baseline SCT and ratio of SCT (after increased retinal haemorrhages was observed) to baseline SCT, baseline spherical equivalent, and occlusion distance from the optic disc margin did not significantly differ between groups (*p* > 0.05, Table 5).The best vision achieved before and after the increased retinal haemorrhages were observed was significantly worse in the ≥ 50% group (0.24 ± 0.19 versus 0.39 ± 0.21 logMAR, *p* = 0.007, Table 6); this was not observed in the < 50% group (0.29 ± 0.19 versus 0.35 ± 0.18 logMAR, *p* = 0.145, Table 6).

**Table 4:** Differences in demographic characteristics between the < 50% and ≥ 50% increased retinal haemorrhages groups in branch retinal vein occlusion patients.

**Parameters**	** < 50% increase in retinal haemorrhages group** **(n = 11)**	** ≥ 50% increase in retinal haemorrhages group** **(n = 11)**	***P value***
Age in years, mean (SD)	64 (8)	70 (11)	0.207
Sex, n (%) male	6 (54.5%)	4 (36.4%)	0.392
Laterality, n (%) right eye	8 (72.7%)	6 (54.5%)	0.659
Diabetes, n (%)	2 (18.2%)	2 (18.2%)	1.000
Hypertension, n (%)	3 (27.3%)	3 (27.3%)	1.000
Hyperlipidaemia, n (%)	1 (9.1%)	1 (9.1%)	1.000

**Table 5:** Comparison of clinical characteristics between the < 50% and ≥ 50% increase in retinal haemorrhages groups in branch retinal vein occlusion patients.

**Parameters**	** < 50% increase in retinal haemorrhages group** **(n = 11)**	** ≥ 50% increase in retinal haemorrhages group** **(n = 11)**	***P value***
Area, superior, n (%)	7 (63.6%)	6 (54.5%)	1.000
Type of BRVO, ischaemic type, n (%)	5 (45.5%)	7 (63.6%)	0.685
Type of BRVO, macular, n (%)	1 (9.1%)	4 (36.4%)	0.311
Initial therapy, bevacizumab, n (%)	11 (100.0%)	8 (72.7%)	0.214
Baseline best corrected visual acuity, logMAR (SD)	0.60 (0.23)	0.88 (0.15)	0.015*
Baseline intraocular pressure, mmHg, mean (SD)	14.64 (3.17)	14.62 (2.50)	0.733
Baseline spherical equivalent, mean (SD)	1.32 (0.81)	2.17 (3.11)	0.842
Baseline central retinal thickness, μm, mean (SD)	461.82 (106.17)	561.46 (69.89)	0.013*
Baseline subfoveal choroidal thickness, μm, mean (SD)	230.45 (74.97)	180.69 (84.43)	0.082
Occlusion distance from optic disc margin, μm, mean (SD)	899.64 (372.0)	653.15 (590.03)	0.072
Amount of increased retinal haemorrhage, %, mean (SD)	10.18 (7.32)	73.76 (13.37)	0.000*
Increased retinal haemorrhage period from the baseline, month, mean (SD)	5 (2)	7 (2)	0.088
Increased retinal haemorrhage period after last injection, days, mean (SD)	59.27 (10.53)	103.27 (26.85)	0.000*
Central subfield macular thickness compared to baseline, %, mean (SD)	66.02 (13.57)	78.03 (13.30)	0.019*
Subfoveal choroidal thickness compared to baseline, %, mean (SD)	97.84 (4.76)	100.80 (3.66)	0.116

*BRVO* branch retinal vein occlusion

*Statistically significant as calculated using the Mann–Whitney U test or Fisher’s exact test.

**Table 6:** Comparison of best vision achieved between before and after increase in retinal haemorrhages (≥ 50% versus < 50%) in branch retinal vein occlusion patients.

**Parameters**	**Before increase in retinal haemorrhages**	**After increase in retinal haemorrhages**	***P value***
Best corrected visual acuity in the ≥ 50% group, logMAR, mean (SD)	0.24 (0.19)	0.39 (0.21)	0.007*
Best corrected visual acuity in the < 50% group, logMAR, mean (SD)	0.29 (0.19)	0.35 (0.18)	0.145

*Statistically significant as calculated using Wilcoxon signed rank test.

## Discussion

The current study retrospectively analysed the baseline characteristics of BRVO patients treated for MO, and subgroup analysis was performed based on the amount by which the retinal haemorrhages increased. At baseline, the increased retinal haemorrhages group was older than the non-increased retinal haemorrhages group. Young et al. ^14^ reported that the veins stiffen with age, which could lead to higher intraluminal pressureassociated with an increasedrisk of retinal haemorrhage in BRVO patients. The higher baseline CSMT with the higher proportion of ischaemic type BRVO seen in the increased retinal haemorrhages group implies that the severity at the time of presentation is related to increased retinal haemorrhages during MO treatment. A shorter occlusion distance from the optic disc margin was also found in the increased retinal haemorrhage group. However, the ischaemic type of BRVO was the only factor that was associated with increased retinal haemorrhage in the multivariate analysis.

The subgroup analysis revealed that the ≥ 50% group exhibited significantly higher baseline CSMT and poorer baseline BCVA values. Moreover, the comparison of the best vision achieved before and after an increase in retinal haemorrhage was significantly worse in the ≥ 50% group. Bayat et al. ^15^. reported that the baseline BCVA is a predictor of visual gain, while baseline CSMT is a predictor of anatomical gain in BRVO patients treated with intravitreal ranibizumab injection for MO. In addition, our subgroup analysis highlighted that the poor visual prognosis in those with low baseline BCVA and CSMT may have resulted from a ≥ 50% increase in retinal haemorrhages during the MO treatment. The shorter mean period between the last intravitreal injection and the increased retinal haemorrhages was observed in the < 50% group. It is known that the single intravitreal bevacizumab injection for BRVO induced ME lasts for 6 weeks to 2 months^16,17^. Interestingly, most patients were treated with intravitreal bevacizumab injection in this study, and the mean period was 59.27 ± 10.53 days in the < 50% group. The lower CSMT ratio (date of increased retinal haemorrhages compared to baseline) was also observed in this group. One previous study hypothesized that bevacizumab reduces VEGF-mediated permeability and steroids reduce inflammation and limit extravasation from the blood vessels^18^. From these observations, we suggest that the < 50% increased retinal haemorrhage resulted from an increase in VEGF-mediated permeability as the effect of intravitreal bevacizumab decreases. On the contrary, the longer period between the last intravitreal injection and the increased retinal haemorrhages with the higher CSMT ratio (date of increased retinal haemorrhages compared to baseline) might have resulted from other causes. One previous report demonstrated a rebound effect after intravitreal bevacizumab injection and a recurrence of BRVO with massive retinal haemorrhages^19^. As bevacizumab cannot prevent the recurrent occlusion of BRVO, we believe that the recurrent occlusion might be related to the ≥ 50% increase in retinal haemorrhages. Previous studies reported a BRVO recurrence rate of 3.3–14.4%^20,21^. In our study, however, 16.2% patients showed ≥ 50% increased retinal haemorrhages. This is because we evaluated MO-treated BRVO patients and assessed retinal haemorrhage changes that were limited to the primary area of BRVO. We hypothesis that the small increase in retinal haemorrhages was related to the increase in VEGF-mediated vascular permeability, while the large increase in retinal haemorrhages was related to the recurrent occlusion in the first BRVO. Further studies with FA at the time of increased retinal haemorrhages will be beneficial.

The current study had some limitations. First, the retrospective design may have resulted in a patient selection bias. Second, the increased retinal haemorrhages were first detected by the human eye, so patients with minimal increases of retinal haemorrhages may have been overlooked. Third, this study included only a small number of patients in subgroup analysis. Fourth, the colour images of the fundus obtained from the OCT did not provide wide-field images. Retinal haemorrhages beyond the colour images were not measured in this study. One previous study demonstrated that most BRVO (particularly major BRVO) were ischaeminc in nature^22^. Therefore, further studies measuring the quantitative amount of ischaemia in the wide-field-based 7-standard field images might provide more information. Finally, we measured the amount of retinal haemorrhages within a given 2-dimensional area, so any changes in the third dimensions would have been overlooked and the actual amount of retinal haemorrhages may have been underestimated. However, we believe that this is the first study to assess the clinical characteristics of increased retinal haemorrhages during MO treatment in cases of BRVO.

In conclusion, the ischaemic type of BRVO is related to increased retinal haemorrhages during the treatment of MO in those with BRVO. Among the patients who experienced an increase in retinal haemorrhages, an increase of ≥ 50% was associated with higher CSMT and poorer BCVA values at baseline, as well as poorer visual gain.

## Data Availability

The datasets generated and/or analysed during the present study are available from the corresponding author on reasonable request.
